# The turnover intentions and intentions to leave the country of foreign-born physicians in Finland: a cross-sectional questionnaire study

**DOI:** 10.1186/s12913-019-4487-1

**Published:** 2019-09-03

**Authors:** Tarja Heponiemi, Laura Hietapakka, Anu Kaihlanen, Anna-Mari Aalto

**Affiliations:** National Institute for Health and Welfare, P.O. Box 30, 00271 Helsinki, Finland

**Keywords:** Migration, Health care workforce, Turnover, Foreign-born physicians

## Abstract

**Background:**

A physician shortage is a worldwide problem and foreign-born physicians fill in the shortage of physicians in many developed countries. One problem that is associated with the physician shortage is increased physician turnover. Also, regarding foreign-born physicians, migration can be costly. The present study aimed to examine the turnover intentions and intentions to leave the country of foreign-born physicians. We examined how demographics, discrimination, language problems, perceived employment barriers, satisfaction with living in Finland, team climate, job satisfaction and patient-related stress were associated with these factors.

**Methods:**

The present study was a cross-sectional questionnaire study among 371 foreign-born physicians in Finland that were aged between 26 and 65 (65% women). Binary logistic regression analyses were conducted to examine the associations.

**Results:**

Half of the respondents had turnover intentions and 14.5% had considered leaving the country. High satisfaction with living in Finland was associated with a lower likelihood of both turnover intentions and intentions to leave the country. High levels of discrimination and employment barriers were associated with a high likelihood of turnover intentions whereas good team climate was associated with a low likelihood of turnover intentions. High levels of language problems were associated with a high likelihood of intentions to leave the country.

**Conclusions:**

The present study showed the importance of satisfaction with living in the host country, the prevention of discrimination and employment barriers, language skills and a good team climate for the retention of foreign-born physicians in their current job and in the host country. Thus, to keep their foreign-born physicians, health care organisations should implement measures to tackle these challenges. Organisations could arrange, for example, diversity training, self-assessment, team reflections, leadership coaching and culturally-specific networks. Moreover, internships associated with the qualification process could be utilised better in order to give a thorough introduction to the host country’s health care environment and the possibilities for learning the language.

**Electronic supplementary material:**

The online version of this article (10.1186/s12913-019-4487-1) contains supplementary material, which is available to authorized users.

## Background

Physician shortage is an increasing problem that threatens the functioning of the health care sector worldwide. One problem associated with the physician shortage is increased physician turnover. For example, one-fifth of English general practitioners (GPs) have been found to intend to quit direct patient care within 5 years [1]. This is worrying, given that turnover intention has a direct association with actual turnover and is regarded as an important predictor of quitting [2–5]. Physician turnover may lead to decreased productivity, poorer patient care and to an increased need to recruit and train new physicians [6]. This can be costly and affect health outcomes [6]. It has been shown that work-related factors such as better collegial support and opportunities for professional development may help to recruit and retain physicians [7]. Whereas, low job satisfaction, work-related stress, physical violence or threats from patients, and dealing with uncooperative patients have all been associated with higher levels of physicians’ turnover intentions [8–12].

Foreign-born physicians fill in the shortage of physicians in many developed countries, such as the US, the UK, Australia and Canada [13]. In Finland, the number of foreign-born physicians has not traditionally been very high, but since the year 2000 the number has doubled, being 4 % of the physician population in 2000 and 8.4% in 2012 [14, 15]. Finland joining the EU in 1995, a change in Finnish policy environment, and increasing physician shortage have been suggested as contributing to inflow increase since 2000 [14]. Finland has alleviated the shortage of practitioners, especially general practitioners, with foreign-born physicians, but it has been suggested that Finland may have difficulties in attracting and retaining foreign-born physicians in the future, for example, due to difficult licensing processes and high job strain in primary care [14].

According to the dual labour market theory [16], migrant employees have a high risk of job instability and high turnover. Previous results show that foreign-born physicians are more likely to have turnover intentions compared to native physicians [11, 17]. Foreign-born physicians’ turnover may be very harmful for organizations, given that the costs of turnover may be even more severe because of the higher investments in training and support needed among foreign-born physicians compared to native physicians.

Health care organisations have to put effort into integrating foreign-born physicians into the work environment of the country in question and all this effort is lost if the physicians leave the country. Therefore, another aspect of interest that might be of importance is foreign-born physicians’ intention to leave the country. It has been suggested that physician migration is a ‘carousel’, where physicians move from one country to the next, trying to find further advantages or as a response to disappointments in the host country [18]. In a study from Ireland, it was found that 23% of foreign physicians planned to return home and 47% planned to migrate onwards; low satisfaction with living in Ireland was associated with high migratory intentions [19]. Low job satisfaction, long working hours and poor working conditions have also been mentioned as factors that are associated with the intent to leave the country [20, 21].

For foreign-born physicians, also the success of integration into working life is likely an important factor when deciding whether to change job or to stay in the host country. Previous studies have shown that there are notable problems in such integration. Foreign-born physicians have been found to be susceptible to encountering insensitivity, discrimination and isolation in their workplace [22, 23]. Moreover, foreign-born physicians have also been found to encounter threats or violence from patients [11] and harassment from colleagues [24]. Also high work-related distress and the lack of professional support and consultation possibilities may burden foreign-born physicians more than native physicians [25]. Foreign-born physicians may face many other barriers to their employment also, one of the most obvious ones being poor language skills regarding the host country’s language [26, 27]. The importance of foreign-born physicians’ language problems in burdening the whole work team has been highlighted in a recent Finnish study [27]. Language problems have high relevance to the quality of health care given that it may lead to diagnostic errors and inappropriate treatment [28] as well as result in other communication problems. Moreover, doubts about one’s professional competence norms and legislation may all challenge migrant physicians’ employment [29].

A concept that has previously been found important in culturally diverse working environments is that of *team climate* [30]. A good team climate in a work unit might leave less space for discrimination and promote good cross-cultural communication, and therefore, it might help to decrease foreign-born physicians’ turnover intentions.

Thus, foreign-born physicians’ turnover and emigration may have many negative ramifications for health care organizations. Moreover, previous studies show that work-related factors such as job satisfaction have an effect on physicians’ turnover intentions [8–12]. In addition, migration-related variables, like discrimination, length of stay and language problems, have been found to be important aspects of foreign-born physicians’ integration to working life [25]. However, evidence about factors associated with foreign-born physicians’ intentions to turnover and country change is still lacking. In light of these findings, the present study aimed to examine the turnover intentions and intentions to leave the country of foreign-born physicians in Finland. More specifically, we examined how gender, age, employment sector, country of birth, the number of years from getting a practicing license in Finland, discrimination, language problems, perceived employment barriers, satisfaction with living in Finland, team climate, job satisfaction and patient-related stress were associated with turnover intentions and intentions to leave the country. Gender, age and employment sector were chosen as adjustments variables because they have been found to have a big adjusting effect among physicians on relations between work characteristics and different outcomes in previous studies with Finnish physicians [31–33].

## Methods

### Study sample

A questionnaire survey was conducted in the autumn of 2017 (14th of September–5th of November) in Finland. Information was obtained from Valvira’s (the National Supervisory Authority for Welfare and Health) Terhikki central register. All the physicians born after 1944 who had completed their medical education outside of Finland and who had received a license to practice medicine in Finland (*n* = 1564) were included to the sample. Email addresses (provided by the Finnish Medical Association) or postal addresses (provided by the Population Register Centre) were obtained for 1012 physicians. Living abroad or not having a permanent address in Finland and non-disclosure for purposes such as direct advertising or for personal safety reasons were the most important reasons for not getting the postal addresses. The physicians whose mother tongue was one of the two official language in Finland (Finnish or Swedish; *n* = 203) were excluded to rule out native Finns who had studied abroad. Consequently, the questionnaire was sent to 809 physicians.

We used webropol questionnaire service and sent an email invitation with a link to the electronic survey for those whose email addresses we had. For others, a postal invitation to participate in the electronic survey was sent to their home addresses. Two reminders in 3 weeks interval were sent to those who had not responded. Additional postal paper version of the questionnaire (only in Finnish) was sent with the second reminder. Altogether 375 (46% of those who the questionnaire was sent to) physicians responded to the survey. The electronic questionnaire was available in Finnish, Swedish, Estonian, Russian and English languages; 153 of the respondents answered the electronic version in Finnish, two in Swedish, 64 in Estonian, 60 in Russian and 22 in English. Seventy-four responded by the postal questionnaire, which was only available in Finnish. We asked the country of birth in the questionnaire, thus we were able to identify those who were actually foreign-born. Of the respondents, we excluded two respondents who had studied abroad and whose mother tongue was other than Finnish or Swedish but who reported being born in Finland. Moreover, two respondents were excluded because they did not provide us with the information about their country of birth. Thus, the final study sample included 371 foreign-born physicians aged between 26 and 65 (mean age: 39.1; *SD* = 9.09; 65% women).

Ethical approval was obtained from the ethics committee of the National Institute for Health and Welfare in Finland.

### Measurements

Most of the measures used in the present study have already been tested in our previous data gathering among Finnish foreign-born physicians in 2010/2011 [30]. The questionnaire can be found from the Additional file 1.

*Turnover intentions* were measured with a question asking whether the respondent had planned changing employment. The response options were: 1) *no*, 2) *possibly*, 3) *yes*. For the analyses the measure was coded as 0 = *no* and 1 = *yes* and *possibly*. *Intentions to move country* was measured with a question asking whether the respondent had plans to move to work in another country during the next 12 months. The response options and coding were the same as in the question for turnover intentions. Binary coding was chosen for these variables because the amount of “yes” answers was rather low especially for intention to leave the country (4,3%) and binary coding allowed us to conduct logistic regression analyses (with odds ratios and easy interpretation of the results).

*Discrimination* was measured with a mean of three items (α = 0.72) that have previously been used among foreign-born physicians in Finland [34] to assess whether a person had experienced personal discrimination at work during the last 12 months coming from 1) superiors and management, 2) colleagues and fellow employees and 3) patients/clients. The items were rated on a five-point Likert scale ranging from 1 (*hardly ever*) to 5 (*very often* or *continuously*).

*Language problems* were measured with the mean of seven items (α = 0.96) that have previously been used among foreign-born physicians in Finland [34] to ask how often the respondent experienced work-related communication challenges in using Finnish language in a) face-to-face communication with patients, b) face-to-face communication with co-workers, c) workplace meetings, d) keeping up with professional literature, e) recording data in patient information systems, f) electronic communication and g) communication over the phone. The items were rated on a five-point Likert scale ranging from 1 (*hardly ever*) to 5 (*very often* or *continuously*).

*Employment barriers* were measured with the mean of four items (α = 0.70) that asked whether employer attitudes, difficulties in finding information about job vacancies, insufficient relationships with people of Finnish origin and one’s uncertainty had prevented the respondent from getting a job she or he wanted in Finland during the past 2 years (1 = *not at all*, 2 = *to some extent* and 3 = *a lot*).

*Satisfaction with living in Finland* was assessed by a asking how satisfied or dissatisfied the respondent was overall with his or her life in Finland. The response options ranged between 0 (*very dissatisfied*) and 10 (*very satisfied*). For the statistical analyses, the measure was coded as *low* = 0–7 (26% of the respondents) and *high* = 8–10 (74%) (this was done because the measure included only one item).

*Team climate* was measured with the mean of four items (α = 0.90) from the Team Climate Inventory (TCI) [35] which measures team participation, such as interaction frequency and information sharing. The items were rated on a five-point Likert scale, ranging from 1 (*fully disagree*) to 5 (*fully agree*), with higher scores indicating a better team climate.

*Job satisfaction* was measured with one item from Job Diagnostic Survey’s [36] scale for overall satisfaction with the job (‘Generally speaking I am very satisfied with this job’). The item was rated on a five-point Likert scale ranging from 1 (*fully disagree*) to 5 (*fully agree*). Because this measure included only one item this measure was coded as *low* = 1–4 (54% of the respondents) and *high* = 5 (46%) for the analyses.

*Patient-related stress* was measured by the mean of three items (α = 0.82) that asked participants how often the situation described in the statement had disturbed them, worried them or caused them stress during last half-year period using a five-point Likert scale ranging from 1 (*hardly ever*) to 5 (*very often* or *continuously*) with higher scores indicating higher stress. The items were: a) ‘Patients’ expectations frequently differ from those of health care personnel’, b) ‘Difficult patients who complain, blame and criticize’ and c) ‘Patients who are unwilling to co-operate and passive’. These items have been developed based on earlier studies carried out in the health care sector [37, 38]. The measure has been used previously among foreign-born physicians [25].

*The employment sector* was categorised into three groups: the municipal sector (e.g. primary care), the state sector (e.g. hospitals) and the private sector. *The country of birth* was asked about in the questionnaire with an open-ended question and categorised as a) Estonia, b) the Russian Federation / former Soviet Union, c) EU/EEA countries other than Estonia and d) other countries not covered by a–c.

### Statistical analysis

The effects of independent variables on turnover intentions and intentions to leave the country were examined with binary logistic regression analyses (in separate analyses). The analyses were conducted in two steps. First, the effects of gender, age, employment sector, country of birth and the number of years from getting a practicing license in Finland were examined (Model A). In the second step, discrimination, language problems, employment barriers, satisfaction with living in Finland, team climate, job satisfaction and patient-related stress were added to the former model (Model B). A multi-collinearity analysis of the study variables was performed. The variance inflation factors (VIF) associated with the variables were all below 1.5, showing that multi-collinearity is not a concern in this study [39].

All analyses were performed using SPSS software, version 25.0.

## Results

The characteristics of the study population can be found in Table 1. There were slightly more female respondents than male respondents and the most common working sector was the municipal sector. Most of the respondents came from Estonia (38%) or the Russian Federation / former Soviet Union (30%). The mean time since getting a license to practice a profession in Finland was 5.2 years. Almost half of the respondents had turnover intentions and 14.5% had considered leaving the country. Seventy-four percent were satisfied with living in Finland (gaining a rating of 8–10 on a scale ranging from 0 to 10; the mean score was 8.3).

**Table 1:** The characteristics of the study sample

	n	%
Gender		
Woman	240	65.2
Sector		
Municipal (primary care)	280	76.5
State (hospitals)	34	9.3
Private	52	14.2
Country of birth		
Estonia	142	38.3
Russian federation/former Soviet Union	111	29.8
Other EU/EEA country	64	17.3
Other country	54	14.6
Turnover intentions		
Yes	186	50.8
Intentions to change the country		
Yes	53	14.5
Satisfaction with living in Finland		
Low	96	25.9
Job satisfaction		
Low	201	54.3
	Mean	SD
Age (26–65)	39.1	9.13
Years from getting practicing license in Finland	5.24	3.10
Discrimination (1–5)	1.56	0.70
Language problems (1–5)	2.03	1.20
Employment barriers (1–3)	1.43	0.45
Team climate (1–5)	4.16	0.83
Patient-related stress (1–5)	2.41	0.82

Logistic regression analyses showed that discrimination, employment barriers, satisfaction with living in Finland and team climate were significantly associated with turnover intentions in fully adjusted model (Table 2). High levels of discrimination and employment barriers were associated with a higher likelihood of turnover intentions. Those who had high satisfaction regarding living in Finland were about half less likely to have turnover intentions compared to those with low satisfaction. High levels of team climate were associated with a lower likelihood of turnover intentions. To be able to evaluate the relative importance of these significant variables, we calculated standardized beta weights for them according to King’s [40] instructions. Standardized beta weights were 0.086 for discrimination, 0.085 for employment barriers, − 0.068 for satisfaction with living in Finland and − 0.086 for team climate. Thus, satisfaction with living in Finland had lower power compared to other variables.

**Table 2:** The results of logistic regression analysis for turnover intentions, odds ratios (ORs) and 95% CIs

	Model A^a^			Model B^b^		
	OR	(95% CI)	*p*	OR	(95% CI)	*p*
Gender			0.917			0.746
Man	1			1		
Woman	1.02	0.65–1.61		1.09	0.66–1.80	
Age	0.99	0.78–1.24	0.904	0.93	0.71–1.21	0.566
Employment sector			0.356			0.384
Municipal (e.g., primary care)	1			1		
State (e.g., hospitals)	1.66	0.78–3.54		1.68	0.74–3.82	
Private	0.88	0.47–1.63		0.86	0.43–1.72	
Country of birth			0.259			0.051
Estonia	1			1		
Russia/former Soviet Union	0.79	0.46–1.36		0.77	0.42–1.42	
Other EU/EEA	1.12	0.60–2.10		0.70	0.34–1.45	
Other	0.54	0.27–1.09		0.30	0.13–0.70	
Years from Finnish practicing license	0.91	0.72–1.15	0.452	0.93	0.72–1.20	0.580
Discrimination				1.64	1.09–2.48	0.018
Language problems				1.16	0.94–1.42	0.175
Employment barriers				2.13	1.13–4.01	0.019
Satisfaction with living in Finland						0.047
Low				1		
High				0.54	0.29–0.98	
Team climate				0.71	0.53–0.95	0.024
Job satisfaction						0.605
Low				1		
High				0.87	0.51–1.48	
Patient-related stress				0.97	0.70–1.33	0.830

Continuous variables were used as continuous standardized variables and the model ORs presented indicate the likelihood of passing from no turnover intentions to having turnover intentions, compared to one standard deviation change in continuous independent variables

*OR* Odds ratio, *CI* Confidence interval

^a^Adjusted for gender, age, employment sector, country of birth, and years from getting practicing licence in Finland

^b^Additionally adjusted for discrimination, language problems, employment barriers, satisfaction with living in Finland, team climate, job satisfaction, and patient-related stress

Logistic regression analyses showed that language problems and satisfaction with living in Finland were significantly associated with intentions to leave the country in fully adjusted model (Table 3). High levels of language problems were associated with a higher likelihood of intentions to leave the country. Those who had high satisfaction regarding living in Finland were 0.37 times less likely to have intentions to leave the country compared to those with low satisfaction. Standardized beta weights were − 0.054 for satisfaction for living in Finland and 0.050 for language problems. Thus, there were no differences between these two variables, but beta weights showed that our studied variables had more power in relation to turnover intentions than intentions to leave the country.

**Table 3:** The results of logistic regression analysis for intentions to leave the country, odds ratios (ORs) and 95% CIs

	Model A^a^			Model B^b^		
	OR	(95% CI)	p	OR	(95% CI)	p
Gender			0.181			0.239
Man	1			1		
Woman	0.65	0.35–1.22		0.65	0.32–1.33	
Age	0.85	0.60–1.20	0.345	0.77	0.52–1.14	0.194
Employment sector			0.043			0.115
Municipal (e.g., primary care)	1			1		
State (e.g., hospitals)	3.15	1.26–7.87		2.90	1.05–7.97	
Private	1.00	0.40–2.47		1.05	0.40–2.80	
Country of birth			0.119			0.385
Estonia	1			1		
Russia/former Soviet Union	0.44	0.18–1.09		0.42	0.16–1.15	
Other EU/EEA	1.32	0.58–3.03		0.80	0.30–2.11	
Other	1.34	0.54–3.31		0.88	0.30–2.53	
Years from Finnish practicing license	1.05	0.74–1.50	0.787	1.17	0.80–1.70	0.419
Discrimination				1.19	0.71–2.00	0.505
Language problems				1.36	1.01–1.83	0.046
Employment barriers				1.94	0.93–4.06	0.077
Satisfaction with living in Finland						0.009
Low				1		
High				0.37	0.18–0.78	
Team climate				0.79	0.56–1.11	0.172
Job satisfaction						0.959
Low				1		
High				1.02	0.46–2.29	
Patient-related stress				1.08	0.69–1.68	0.736

Continuous variables were used as continuous standardized variables and the model ORs presented indicate the likelihood of passing from no intentions to leave the country to having intentions to leave the country, compared to one standard deviation change in continuous independent variables

*OR* Odds ratio, *CI* Confidence interval

^a^Adjusted for gender, age, employment sector, country of birth, and years from getting practicing licence in Finland

^b^Additionally adjusted for discrimination, language problems, employment barriers, satisfaction with living in Finland, team climate, job satisfaction, and patient-related stress

## Discussion

The present study examined foreign-born physicians’ turnover intentions and intention to leave Finland. Our results showed that those who had high satisfaction regarding living in Finland were less likely to have turnover intentions and intentions to leave the country compared to those with low satisfaction regarding living in Finland. Moreover, high levels of discrimination and employment barriers were associated with a higher likelihood of turnover intentions, and high levels of language problems were associated with a higher likelihood of intentions to leave the country. In contrast, high levels of team climate were associated with a lower likelihood of turnover intentions.

Our finding that satisfaction with living in Finland was associated with both turnover intentions and intentions to leave the country is congruent with previous findings. For example, in Ireland almost half of the foreign-born physicians planned to migrate onwards and dissatisfaction with living in Ireland was associated with these migratory intentions [19]. Among Canadian family physicians, dissatisfaction with professional life was the most powerful predictor of planning migration abroad and interprovincial migration [41]. Thus, satisfaction with living in the host country, professional life and work–life balance are important factors that attach foreign-born physicians to their workplaces and host country. Fortunately, a previous study showed that foreign-born physicians had better life satisfaction compared to those physicians who remained in their source country and even compared to native physicians in the host country [42].

The present study found that discrimination was associated with turnover intentions but not with intentions to leave the country. This is logical given that the present study measured work-related discrimination coming from management, colleagues and patients, and thus, the discrimination is experienced more as a problem with the current job, not as a problem with the host country. Perceived discrimination (also) outside work environment would perhaps associate better with intentions to leave the country. Thus, to retain their foreign-born physicians, health care organisations should focus on eliminating discrimination in the workplace. For example, diversity training; resource development and provision; systems to monitor staff and client outcomes including self-assessment; flexible working arrangements and new culturally-specific networks have been suggested as means to reduce culture-based discrimination in the workplaces [43]. However, it seems that there is still a lot to do in this regard given that foreign-born physicians have been found to encounter insensitivity, discrimination and isolation in their workplaces [22, 23]. Moreover, foreign-born physicians face threats or violence from patients [11], harassment from their colleagues [24] and difficulties in interpersonal interactions [29]. Physicians and nurses with African origin seem to be especially vulnerable to discrimination in Europe [34, 44].

A major problem among foreign-born physicians is poor language skills in regard to host country’s mother tongue [26], which has come up also in Finland [34], and according to our results, this also pushes foreign-born physician to move away from Finland. Language problems may make physicians’ work in a foreign country very difficult as it may lead to diagnostic errors and inappropriate treatment, as well as result in communication problems [28]. It has been found that foreign-born physicians’ language problems burden work teams and increase responsibility in patient care, especially that of nursing personnel [45]. Foreign-born physicians’ language problems have been found to be most evident within face-to-face doctor–patient conversations and doctor-patient consultations on the telephone [45].

We found that those who experienced that employment barriers – such as employer attitudes, insufficient relationships with people of Finnish origin and one’s uncertainty – hindered them from getting a job were more eager to change their current job. Thus, it is possible that those who experienced difficulties in getting employed were more likely to be in jobs that were not ideal for them. Previous studies show that migrant employees face discrimination in occupational attainment and often have to work in lower-level jobs [29, 46, 47]. Migrated physicians coming from African countries have been found to experience de-skilling, problems in the recognition of their qualifications and the need for extra training in Europe [44].

According to our results, foreign-born physicians have less turnover intentions in workplaces where the team climate is good. Thus promoting a good team climate in cross-cultural work teams may help retain foreign-born physicians. For maintaining good team climate in multicultural teams organizations should focus on management of communication, putting emphasis on shared universal values, offering regular team reflections, decreasing time constraints, and giving formal leadership coaching [48]. For leaders it is important to focus on role-modelling civil engagement, reflections and willingness to receive feedback, allowing for minority opinions and avoiding blame and criticism [48]. The conflict management is complex in multicultural teams [49] and it has been previously shown that a poor team climate is especially a risk in culturally diverse work units [30], thus it is also important to recognize the role of emotions and how to use emotions in facilitating team processes [50]. A good team climate may also promote high quality care because it may increase professional support and shared objectives [51, 52]. A good team climate promotes free communication, information sharing and experiences of acceptance [35, 53].

Surprisingly job satisfaction and patient-related stress were not associated with turnover intentions or intentions to leave the country in our study. This is congruent with a previous finding among Swedish foreign-born physicians showing that patient-related stress was not associated with turnover intentions after adjusting for control over work pace and empowering leadership [11]. Instead that study found that actual threats or violence from patients was associated with turnover intentions [11]. Our findings are, however, discrepant to previous studies showing that job satisfaction is associated with turnover intentions, for example, among physicians working in United States [10] and in Guangdong [9]. One reason for the discrepancy may be related to the sample: our sample included only foreign-born physicians, whereas above mentioned studies included mostly native physicians.

Our results show that if countries worldwide wish to keep their foreign-born physicians, they should invest in good work and living conditions, language training and employment possibilities. This is especially important in countries having high numbers of foreign-born physicians such as United States, United Kingdom, Australia and Canada where approximately every fourth physician can be foreign-born [13]. However, in their immigration policies countries should also consider that there can be big negative ramifications and ethical problems in reliance on foreign-born physicians. The emigration can be a great loss to the health systems of the source country which quite often are lower-income countries [13]. There are financial and human capital investments that are lost in the source countries [54]. Therefore, countries with high levels of foreign-born physicians should also consider investing on nation’s own domestic medical education.

### Limitations

The present study relied on self-reported measures, thus inflation of the strengths of relationships and common method variance may have caused problems. Moreover, residual confounding may be an issue here even though we controlled for age, gender, employment sector, country of birth and the number of years from getting a practicing licence in Finland. We cannot draw any causal inferences because of the cross-sectional nature of our study. Moreover, our study has some representative issues because we were not able to get information about citizenship or country of birth from the registers when drawing the sample. Thus, having a mother tongue that is a language other than Finnish or Swedish (the official languages in Finland) was used as an inclusion criterion to attempt to ensure the exclusion of native Finnish physicians who have studied abroad. However, this also excluded foreign-born physicians whose mother tongue was Swedish. Nonetheless, they comprise a very small portion of foreign-born physicians working in Finland (in 2010 they accounted for approximately 2% of foreign-born physicians working in Finland [55]).

Moreover, when interpreting our results it is important to keep in mind that physicians’ intentions to leave will not necessarily translate into action. However, the intention to leave has gained support as an important predictor of actual staff turnover (a typical correlation being 0.50) [5, 56]. Moreover, it has been found that physicians who reported that they were very likely to leave their current practice were 2.4 times more likely to have done so 4 years later than those who reported that they were very unlikely to leave [57]. However, there also exist findings suggesting that intention to leave is not a very powerful indicator of actually leaving, given that only 35% of those who had intended to leave clinical practice had actually left in a three-year follow-up [58]. Another measure of turnover intention than our measure could perhaps be more strongly associated with actual turnover. For example, asking about firm plans to change within the next year or inquiring about moving or getting a new job within the past 3 months might be stronger indicators of actual turnover.

## Conclusions

The present study showed the importance of satisfaction with living in the destination country, the prevention of discrimination and employment barriers, language skills and a good team climate for the retention of foreign-born physicians in their current job and in the host country. Thus, destination countries and health care organisations employing foreign-born professionals should implement programmes addressing these issues. For example, diversity training, self-assessment, team reflections, leadership coaching and culturally-specific networks have been recommended in order to support multicultural work teams [43, 48]. Moreover, continuing training and professional language training are of great importance [45]. Internships associated with the qualification process should be utilised better in order to give both a thorough introduction to the operating environment and the possibility to learn the language of the host country [45].

## Additional file

Additional file 1: Survey questionnaire. Multicultural physicians’ job questionnaire. (PDF 375 kb)

## Data Availability

The datasets used and/or analysed during the current study are available from the corresponding author on reasonable request.

## Abbreviations

CI: Confidence interval
GP: General practitioner
OR: Odds ratio
TCI: Team Climate Inventory
Valvira: The National Supervisory Authority for Welfare and Health
VIF: Variance inflation factor
