# Supplementary material for: The turnover intentions and intentions to leave the country of foreign-born physicians in Finland: a cross-sectional questionnaire study
Source: BMC Health Serv Res. 2019 Sep 3;19:624. doi: 10.1186/s12913-019-4487-1 (PMC6724339; doi:10.1186/s12913-019-4487-1)
Supplement: Supplementary file 1 — Survey questionnaire. Multicultural physicians’ job questionnaire. (PDF 375 kb) [file 12913_2019_4487_MOESM1_ESM.pdf]

MULTICULTURAL PHYSICIAN'S JOB

BACKGROUND INFORMATION

1. Please begin by writing down the ID number in your covering letter *

ID number:

____

4 characters remaining

2. Gender

○ Man

○ Woman

3. Age

____

2 characters remaining

4. What is your mother tongue?

○ Finnish

○ Swedish

○ Estonian

○ Russian

○ German

○ Other, please specify

____

5. What is your marital status?

- ◯ Single
- ◯ Married/Living together
- ◯ Separated/Divorced
- ◯ Widowed

6. If you are married/living together or in a registered partnership, what is your spouse’s mother tongue?

- ◯ Finnish
- ◯ Swedish
- ◯ Estonian
- ◯ Russian
- ◯ German
- ◯ Other, please specify

____

7. Do you have any children living at home with you?

- ◯ No
- ◯ Yes, how many?

____

DEGREE OR QUALIFICATION DETAILS

8. Year for the completion of the basic degree in medicine (Licentiate in Medicine or similar)

____

4 characters remaining

9. Country where you completed your basic degree in medicine(Licentiate in Medicine or similar)

◯ Finland

◯ Sweden

◯ Russia

◯ Estonia

◯ Germany

◯ Other country in the EU/EEA area

◯ Other country outside the EU/EEA area

10. In which year did you receive your license to practice your profession as a self-employed person in Finland?

____

4 characters remaining

11. What kind of licence do you presently hold to exercise your profession?

◯ Self-employed person

◯ Restricted licence (e.g PTL)

◯ Assistant (house officer)

12. Specialisation phase

◯ No specialisation (please skip to the next page)

◯ Specialisation in progress

◯ Specialised

13. Indicate your speciality. If you have rights to several specialities, indicate the most recent one.

◯ Select
◯ Anaesthesiology and intensive care
◯ Surgical specialties
◯ Paediatric
◯ Obstetrics and Gynaecology
◯ Psychiatry
◯ Radiology

◯ Ophthalmology
◯ Internal medicine specialties
◯ Otorhinolaryngology
◯ General Practice
◯ Occupational Health
◯ Neurology
◯ Other fields of specialization

14. Do you have a specialist qualification acquired in Finland?

◯ No

◯ Yes

DETAILS OF MAIN OCCUPATION

While completing it, please rate the questions and statements in the survey on the basis of your current main occupation. If you are not employed at this time, base your answers on your most recent job.

15. Your situation at the moment?

◯ Select
◯ Working
◯ I am full-time retired, participating work-life
◯ I am full-time retired, not participating work-life
◯ I am on maternity/paternity leave
◯ Not working for other reason

16. Employment sector for your main occupation

◯ Municipality/ joint municipal board

◯ State

◯ Private (including University)

17. Workplace for main occupation/working sector

◯ Select
◯ University central hospital

○ Other municipal/joint municipal board hospital
○ Health centre (municipal employment)
○ Health centre (through temporary staffing services)
○ Municipal occupational health care
○ Government agency or institution
○ University
○ Private health clinic or centre, private practice
○ Private occupational health care
○ Foundation, association or organisation
○ Pharmaceutical industry
○ Employment service/temporary staffing agency (physicians employed temporarily in locations other than health centres)
○ Other workplace

18. Occupational title for main occupation

○ Select
○ Medical director, director, chief physician, head of department, deputy chief physician
○ Specialist or senior physician
○ Specializing physician, doctor in training, doctor in special training in general medical practice
○ General practitioner
○ Occupational health physician, chief occupational health physician
○ Professor
○ Clinical lecturer, assistant professor
○ Researcher, assistant
○ Private practitioner
○ Other physicians' job
○ Other than physicians' job

19. Location of main occupation

○ Large city (more than 100,000 citizens)
○ Middle-size city or municipality (20,000–100,000 citizens)
○ Small town or municipality (less than 20,000 citizens)

20. Are you

◯ In permanent employment?

◯ In fixed-term employment?

◯ In private practice?

21. Do you have managerial duties?

◯ No

◯ Yes

22. State whether you main occupation is

◯ Full-time

◯ Part-time, hours/week

____

◯ Partial retirement, working hours/week

____

23. In your opinion, has your foreign origin been an advantage or a disadvantage when looking for a job in Finland?

◯ Select
◯ Advantage
◯ Advantage and disadvantage
◯ Disadvantage
◯ No significance

24. Have you worked in jobs other than physician's jobs in Finland?

◯ Select
◯ No
◯ Less than a year
◯ 1-3 years
◯ 4-10 years
◯ Over 10 years

MULTICULTURALISM AND CULTURAL COMPETENCE

25. Have you received multicultural training?

◯ Select
◯ No
◯ Yes, as part of my studies to become a physician
◯ Yes, after graduating as a physician (e.g. workplace training, other further training)
◯ Yes, I have participated in a project or development work related to multiculturalism

26. How often on average do you meet patients from different cultures in your work?

◯ Select
◯ Never
◯ Daily
◯ Weekly
◯ Monthly
◯ Less than monthly

27. Are there any colleagues of foreign origin in your work community?

◯ Select
◯ None
◯ There are clearly fewer persons of foreign origin than those of Finnish origin
◯ There an equal number or an almost equal number of persons of foreign origin as those of Finnish origin
◯ There are clearly more persons of foreign origin than those of Finnish origin

28. To what extent do you deal with colleagues of foreign origin in your workplace?

◯ Select
◯ Never
◯ Daily
◯ Weekly
◯ Monthly
◯ Less than monthly

29. Please read the following statements associated with multiculturalism. For each statement, select the alternative that best describes your opinion.

	Fully disagree	Somewhat disagree	Neither agree nor disagree	Somewhat agree	Fully agree
I consider it an enrichment to have friendships with people from different cultural backgrounds.	○	○	○	○	○
Cultural diversity is also an enrichment.	○	○	○	○	○
I find it exciting to treat patients with a migration background.	○	○	○	○	○
I consider working in a cross-cultural team an enrichment.	○	○	○	○	○
I enjoy talking to people who have migrated to Finland about their experiences here.	○	○	○	○	○
The interaction with people from other cultural backgrounds helps me reflect upon my own cultural background.	○	○	○	○	○
By communicating with patients with a migration background I can learn about different cultural orientations.	○	○	○	○	○
I would like to make use of training, advising and educational offers, in order to improve my understanding of patients with a migration background.	○	○	○	○	○
It is important for me to treat patients according to their cultural needs and individual values.	○	○	○	○	○
I find it an imposition, when people who migrated to Finland a long time ago, cannot speak Finnish properly.	○	○	○	○	○
People who migrated to Finland should adapt to society, not the other way around.	○	○	○	○	○
Institutions and the public pay too much attention to the special wishes of migrants.	○	○	○	○	○
I have the impression that migrants often assume discrimination, when in fact general rules are simply being enforced.	○	○	○	○	○

30. Please read the following statements associated with multicultural customer/patient work. For each statement, select the alternative that best describes your opinion.

	Fully disagree	Somewhat disagree	Neither agree nor disagree	Somewhat agree	Fully agree
With patients who do not understand Finnish very well, I take more time to explain the treatment options to them.	○	○	○	○	○
In order to achieve the agreed treatment goal, I ask patients with a migration background what they need in terms of support.	○	○	○	○	○
With patients who do not understand Finnish very well, I take more time to discuss their expectations and fears.	○	○	○	○	○
Culturally specific factors of people (e.g. values, behavior norms, beliefs) influence their understanding of disease significantly, and should therefore be assessed and taken into consideration by healthcare professionals.	○	○	○	○	○
I consider the values of patients in relation to family, religion, etc., if they seem relevant for the treatment.	○	○	○	○	○
In my professional interaction with patients with a migration background, I often feel unsure, angry and frustrated.	○	○	○	○	○
I often find it difficult to relate to the elaborations of my patients, when their socio-cultural background is quite different from my own.	○	○	○	○	○
I get impatient when I cannot make myself understood with patients with a migration background.	○	○	○	○	○
I find it difficult to speak slowly in lay language with people who struggle to understand my instructions.	○	○	○	○	○
I prefer treating patients from my own cultural background, than those who seem foreign to me.	○	○	○	○	○
The disease concepts of patients with a migration background are not relevant for treatment success.	○	○	○	○	○
Within the migrant population, there are hardly any differences in terms of health opportunities and disease risks.	○	○	○	○	○

My professional perception, assessment, and behaviour remain untouched by my cultural imprinting.	○	○	○	○	○
The migration experience is a critical life event and can be accompanied by psychosocial stress and health burden.	○	○	○	○	○

WORK AND WORK COMMUNITY

While completing it, please rate the questions and statements in the survey on the basis of your current main occupation. If you are not employed at this time, base your answers on your most recent job. For each statement, select the alternative that best describes your opinion.

31. In the following items, you will be asked to rate the activities of your work unit. Think here of the work unit (department, health center/clinic, small group) that is the most essential to your work.

	Fully disagree	Somewhat disagree	Neither agree nor disagree	Somewhat agree	Fully agree
People keep each other informed about work related issues in the practice	○	○	○	○	○
There are real attempts to share information throughout the practice	○	○	○	○	○
We have a "we are in it together" attitude	○	○	○	○	○
People feel understood and accepted by each other	○	○	○	○	○

32. The following statements refer to procedures in your workplace

	Fully disagree	Somewhat disagree	Neither agree nor disagree	Somewhat agree	Fully agree
Everybody is entitled to express an opinion and their experience if related to matters concerning themselves	○	○	○	○	○
Decisions taken in our workplace have been consistent	○	○	○	○	○
Decisions in our workplace are not biased	○	○	○	○	○

33. The following statements refer to offering and receiving help at your workplace

	Rarely or never	Not often	To some extent	Fairly often	Very often
I receive support and help from my coworkers when I need to	○	○	○	○	○
I receive support and help from my supervisor when I need to	○	○	○	○	○
I help and support my coworkers when they need it	○	○	○	○	○

34. How well do the following statements describe you?

	Fully disagree	Somewhat disagree	Neither agree nor disagree	Somewhat agree	Fully agree
Generally speaking I am very satisfied with this job	○	○	○	○	○
My job requires that I learn new things.	○	○	○	○	○
I get to do a variety of different things in my job.	○	○	○	○	○
I have an opportunity to develop my own special abilities	○	○	○	○	○

35. Discrimination means an unequal treatment of people (without acceptable reason) and placing them in an unfavourable position on the basis of belonging to a certain group. Have you personally experienced discrimination in your workplace in the past 12 months?

	Hardly ever	Not often	Sometimes	Fairly often	Very often or continuously
From superior or management?	○	○	○	○	○
From colleagues/fellow employees?	○	○	○	○	○
From patients/clients?	○	○	○	○	○

36. Intention to change job

	No	Possibly	Yes
Have you planned changing employment?	○	○	○
Have you had plans for moving to work in another country during the next 12 months?	○	○	○

WORKING IN FINLAND

37. Have the following factors prevented you from getting a job you want or complicated getting it or prevented your working in Finland during the past two years?

	Not at all	To some extent	A lot
Lack of language skills (Finnish/Swedish)	○	○	○
Inadequate or unsuitable training (e.g. your previous profession or training is not acknowledged in Finland)	○	○	○
Lack of work experience	○	○	○
Employer attitudes	○	○	○
It is difficult to find information about job vacancies	○	○	○
Insufficient relationships with people of Finnish origin who could help you find a job	○	○	○
Difficulty in finding housing	○	○	○
Your own uncertainty and fear with regard to starting a job	○	○	○
Adherence to a religion or belief	○	○	○
Reconciling work and family life	○	○	○
Other, please specify ________________________________	○	○	○

38. Have the following factors promoted your employment or participation in working life in Finland during the last two years?

	I have not participated	I have participated, but I haven't found it useful	I have participated and found it somewhat useful	I have participated and found it very useful
Integration training	○	○	○	○
Employment services	○	○	○	○
Employer-provided language training	○	○	○	○
Other than employer-provided language training	○	○	○	○
Vocational education at the workplace	○	○	○	○
Vocational education elsewhere than at the workplace	○	○	○	○

Other, please specify ______________________________	○	○	○	○

39. How often on average do you have challenges in communicating in Finnish in the following work-related situations?

	Hardly ever	Not often	Sometimes	Fairly often	Very often or continuously	**I do not need Finnish in my work**
Face-to-face communication with patients	○	○	○	○	○	○
Face-to-face communication with co-workers	○	○	○	○	○	○
Meetings in workplace	○	○	○	○	○	○
Keeping up with professional literature	○	○	○	○	○	○
Recording data in patient information systems	○	○	○	○	○	○
Electronic communication	○	○	○	○	○	○
Communication over the phone	○	○	○	○	○	○

40. To what extent has each of the things listed below disturbed, worried or stressed you in your job in the past six months?

	Hardly ever	Not often	Sometimes	Fairly often	Very often or continuously
Constant rush and pressure due to incompleted work	○	○	○	○	○
Not enough time to perform work properly	○	○	○	○	○
Changes in electronic information systems	○	○	○	○	○
Troublesome, poorly functioning electronic information systems	○	○	○	○	○
Personnel turnover, short-term temps	○	○	○	○	○
Patients’ expectations frequently differ from those of health care personnel	○	○	○	○	○

Difficult patients who complain, blame and criticize	○	○	○	○	○
Patients are unwilling to co-operate and are passive	○	○	○	○	○
Own insufficient professional skills and knowledge	○	○	○	○	○
Responsibility for patients	○	○	○	○	○
Working in a foreign language	○	○	○	○	○
Finnish workplace habits and culture	○	○	○	○	○
Other people do not understand my culture	○	○	○	○	○

LIVING IN FINLAND

In this section, we will ask about your arrival and life in Finland.

41. In which country were you born?

____

42. Are you a Finnish citizen?

○ Yes

○ No

43. Are you living in Finland permanently?

○ Yes

○ No

44. In what year did you (first) move to Finland?

____

45. Overall, how satisfied or dissatisfied are you with your life in Finland?

	0	1	2	3	4	5	6	7	8	9	10	
Very dissatisfied	○	○	○	○	○	○	○	○	○	○	○	Very satisfied

46. On what grounds were you granted a resident permit in Finland?

◯ As an ethnic returnee (e.g., Estonian or Russian)

◯ As the spouse or child of a native of Finland

◯ As the spouse or child of an immigrant residing permanently in Finland

◯ As an employment-based immigrant

◯ As an asylum seeker

◯ As a refugee

◯ Other grounds

HEALTH AND WELLBEING

47. What is your state of health compared to others of your age?

◯ Good

◯ Rather good

◯ Average

◯ Rather poor

◯ Poor

48. Assume that your work ability at its best has a value of 10 and 0 would mean that you could not work at all. How many points would you give to your current work ability?

	0	1	2	3	4	5	6	7	8	9	10	
Very bad	◯	◯	◯	◯	◯	◯	◯	◯	◯	◯	◯	Very good

49. Stress means a situation when a person feels tense, restless, nervous, or anxious, or is unable to sleep at night because his or her mind is troubled all the time. Do you feel that kind of stress these days?

◯ Not at all

◯ Just a little

◯ Some

◯ Quite a lot

○ Very much

50. The following questions relate to your well-being during the past few weeks. Please select the most suitable option for each statement.

	Not at all	Not more than usual	Somewhat more than usual	Much more than usual
Have you recently lost much sleep over worry?	○	○	○	○
Have you recently felt constantly under strain?	○	○	○	○
Have you recently felt you couldn't overcome your difficulties?	○	○	○	○
Have you recently been feeling unhappy and depressed?	○	○	○	○

51. How often during the last four weeks have you had the following symptoms?

	Never	1-3 nights/month	Approximately one night/week	2-4 nights/week	5-6 nights/week	Every night
Having trouble falling asleep	○	○	○	○	○	○
Waking up several times per night	○	○	○	○	○	○
Having trouble staying asleep (including waking up too early)	○	○	○	○	○	○
Waking up after your usual amount of sleep feeling tired and worn out	○	○	○	○	○	○

52. In the space below you can write your notes and opinions about this study and about your experiences of working in Finland. Thank you for your answers!

____

____

____

[Break]

0% completed
